# Fluvastatin Influences Hair Color in C57Bl/6 Mice

**DOI:** 10.3390/ijms140714333

**Published:** 2013-07-10

**Authors:** Ryszard Galus, Krzysztof Włodarski, Jacek Malejczyk, Jarosław JóŸwiak

**Affiliations:** Department of Histology and Embryology, Center for Biostructure, Medical University of Warsaw, Chałubinskiego 5 Str., Warsaw 02-004, Poland; E-Mails: kwlodar@ib.amwaw.edu.pl (K.W.); jacek.malejczyk@wum.edu.pl (J.M.); jaroslaw.jozwiak@gmail.com (J.J.)

**Keywords:** fluvastatin, melanogenesis, melanoma, mice, statins

## Abstract

Our recent *in vitro* experiments suggest that fluvastatin may influence tyrosinase (key enzyme of melanogenesis) synthesis. The aim of the present study was to verify those findings in experiments, *in vitro*, in melanoma cell line, and *in vivo*, in mice. The expression of tyrosinase in B16F10 melanoma cell line, after induction of melanogenesis by UVB irradiation, was examined by Western blot analysis. Afterwards, the effect of fluvastatin on melanin synthesis in hair follicles of C57Bl/6 mice was investigated. The expression of tyrosinase was reduced in the presence of fluvastatin. In mice after anagen induction over the dorsal skin, gel containing fluvastatin in various concentrations was injected subcutaneously, while in part of control groups of mice, gel with placebo was injected. In addition, gel with fluvastatin was injected to four week-old mice (mice in first postnatal anagen) without anagen induction. In extension, injections of gel with fluvastatin or placebo were performed in mice without anagen induction (but after first postnatal anagen). In part of study group of mice (mice after anagen induction and injection of fluvastatin) regrowth of depigmented hair was observed, while in all control groups (mice after injection of placebo), such hair depigmentation over the skin area was not found. In conclusion, this study, for the first time, shows that fluvastatin might affect melanin synthesis *in vivo*.

## 1. Introduction

The 3-hydroxy-3-methylglutaryl coenzyme A (HMG-CoA) reductase inhibitors (statins) represent the most commonly used class of drugs in the treatment of hypercholesterolemia [1]. Experimental evidences suggest a pleiotropic effect of statins [2–9]. Statins may be beneficial in numerous dermatological disorders [10].

Recently, the role of fluvastatin, a frequently used statin, was examined in modulation of tyrosinase *in vitro*, which is a key enzyme of melanogenesis synthesis [11,12]. Since these reports suggest that fluvastatin treatment may influence melanin synthesis, hereby we have verified those findings in consecutive experiments *in vitro* in melanoma cell line and *in vivo* in mice.

First, we have investigated influence of fluvastatin on tyrosinase protein synthesis during melanogenesis enhanced by UVB irradiation of B16F10 melanoma cell line. Afterwards, we have examined influence of fluvastatin on melanin synthesis in hair follicles (HF) of C57Bl/6 mice. Effect of fluvastatin in mice was investigated during intensive hair growth (anagen phase of hair cycle). For control and analysis of potential toxic influence of fluvastatin on hair follicles, statin action was also explored during other phases of hair cycle.

## 2. Results and Discussion

### 2.1. Western Blot

**Figure 1:**
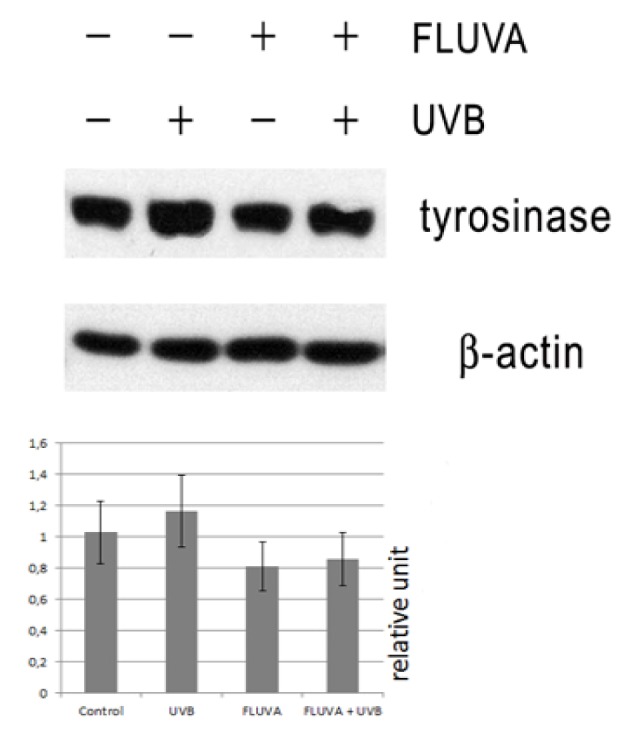
Effect of fluvastatin (FLUVA) and ultraviolet-B irradiation (UVB) on tyrosinase production, as measured by Western blot. β-actin was used as a marker for equal protein loading. The graph presents average levels from four independent Western blots as mean ± SD analyzed using ANOVA test. *p* > 0.05.

Western blot of protein extracts from UVB-irradiated B16F10 melanoma cells demonstrated elevated levels of tyrosinase, compared to non-irradiated cells. Melanoma cells treated only with fluvastatin or irradiated with UVB and treated with 1 μM fluvastatin showed the trend to decrease of tyrosinase, compared to UVB-irradiated cells without fluvastatin. Statistical analysis using ANOVA test did not show significant difference between the levels of tyrosinase from four independent Western blots (*p* = 0.079) (Figure 1).

### 2.2. Macroscopic Examination of Skin and Hair Color

There were no clinical signs of inflammation due to injection of fluva-gel in all concentrations of fluvastatin (fluva-gel 1–3) or placebo-gel in mice; all animals ate well, and were active during the post-injection period.

**Figure 2:**
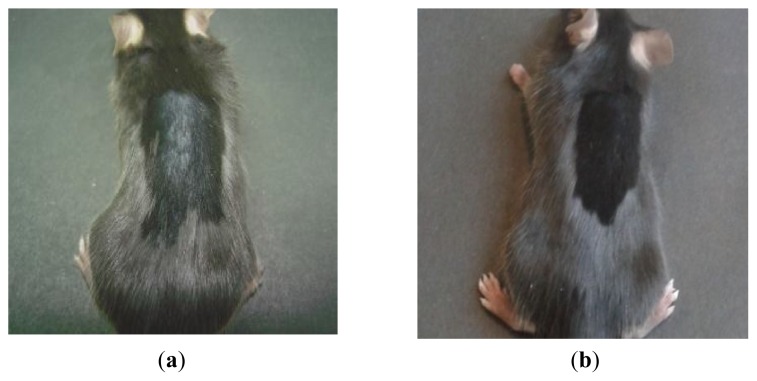
(**a**,**b**) Eight-week-old C57Bl/6 female mice 28 days after anagen induction (performed four weeks after birth) and injection of gel containing fluvastatin (fluva-gel 1) (**a**) and placebo-gel (**b**). Regrowth of light (depigmented) hair on the dorsal skin is observed in (**a**). (**a**) (**b**)

**Figure 3:**
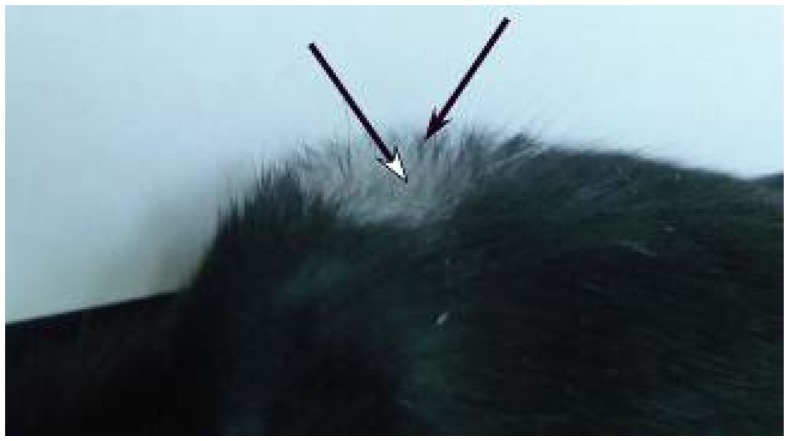
Seven-week-old C57Bl/6 male mice 21 days after anagen induction (performed four weeks after birth) and injection of gel containing fluvastatin (fluva-gel 1). Distal parts of regrowing hair remain black (black arrow), hair beneath is light (white arrow).

**Figure 4:**
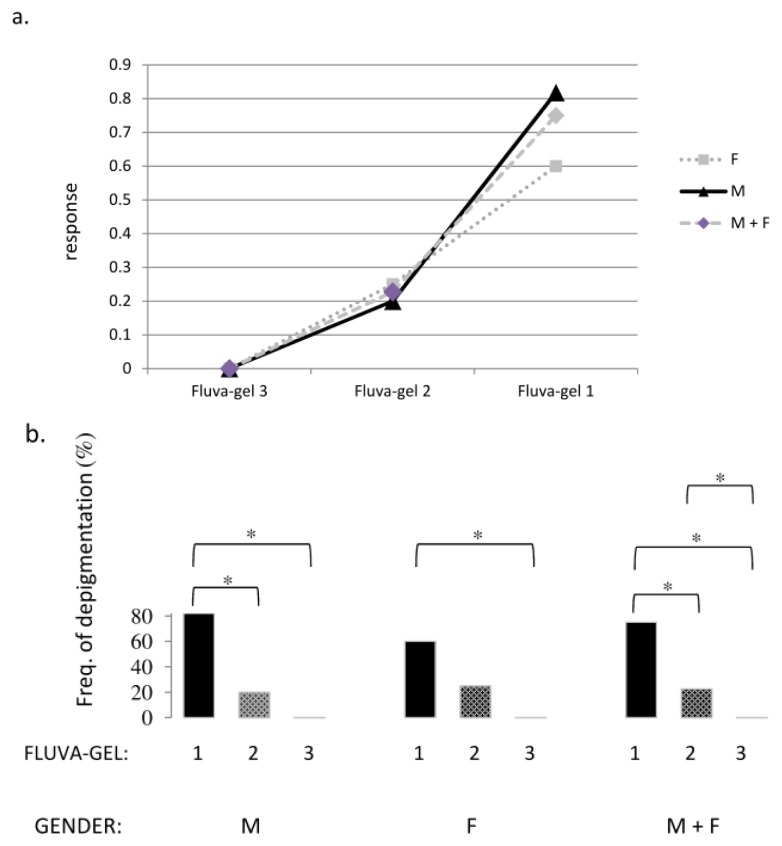
(**a**,**b**) A dose-response curve presents relation between the dose of fluvastatin (fluva-gel 1–3) used in subcutaneous injection and a frequency of depigmentation in mice (M, male, F, female and pooled (M + F)) after anagen induction done during the fourth week of age (**a**). Statistical analysis of differences between the frequencies of hair depigmentation after a single subcutaneous injection of 50 μL of gels with various concentration of fluvastatin (fluva-gel 1–3) in mice after anagen induction performed in fourth week of age (male, female and pooled). * Fisher exact test at *p* < 0.05 (**b**).

In 15 of 24 female and male mice (parts of group 1 and 2 in Table 1), in the skin area where anagen induction and injection of fluva-gel 1 were performed, we observed growth of depigmented hair (Figures 2a and 3), which started 15–17 days after anagen induction. In mice after active hair growth induction and injection of placebo-gel (parts of groups 1 and 2) and also in mice without anagen induction (but after injection of fluva-gel 1 or injection of placebo-gel during sixth and eighth week of age) we observed growth of homogeneous dark black hair (Figure 2b, Table 1: groups 6 and 7). Therefore, hair in the area of anagen induction was shorter when compared to surrounding hair. In mice after anagen induction and injection of fluva-gel with lower concentration of fluvastatin (fluva-gel 2), regrowth of depigmented hair was also seen, although in lower proportion (group 3 in Table 1) than in mice of groups 1 and 2, after fluva-gel 1, while the lowest concentration of fluvastatin (fluva-gel 3) did not provoke depigmentation (group 4 in Table 1). Thus regrowth of depigmented hair is dose-dependent (Figure 4a).

Further observations have proven that depigmentation effect is reversible after the beginning of the next anagen. In our experiments, influence of fluvastatin on hair color after anagen induction is sex-dependent. Effect of fluvastatin is stronger in male than in female mice (Figure 4a): differences between the frequencies of hair depigmentation after a single subcutaneous injection of fluva-gel 1 and fluva-gel 2, as well as between fluva-gel 1 and fluva-gel 3, were statistically significant (in mice after anagen induction performed during the fourth week of age in male mice), while in female mice statistical difference was only between the effect of injection of fluva-gel 1 and fluva-gel 3 (Fisher exact test at *p* < 0.05), as presented in Figure 4b.

The differences in frequencies of depigmentation were the highest in pooled female and male mice (anagen induction in four-week old mice) and were observed between mice after injection of fluva-gel 1, fluva-gel 2 and fluva-gel 3 (statistical difference at *p* < 0.05; Fisher exact test). Calculation in those groups a logistic regression analysis (LRA = 0,098) have shown that probability of depigmentation is tenfold higher in the case of injection fluva-gel 1 than fluva-gel 2.

### 2.3. Microscopic Examination

**Figure 5:**
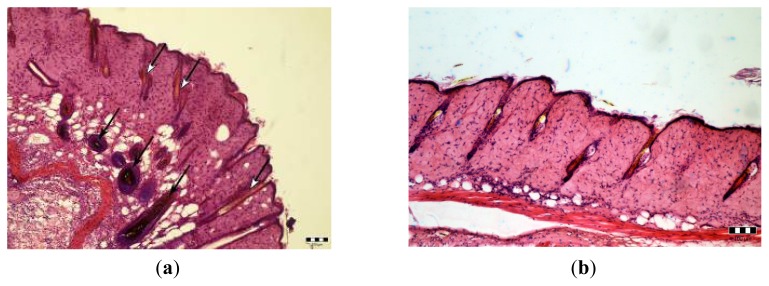
(**a**,**b**) Skin samples collected from male mice 16 days after injection of fluva-gel 1 (**a**) and placebo-gel (**b**); anagen induction was performed in the 4th week after birth. Some hair follicles containing light hair (white arrows) can be found among pigmented hair follicles in anagen (black arrows) (**a**). Dark hair follicles in catagen/telogen (**b**). Hematoxyline-eosin staining. Scale bar: 100 μm.

Skin samples collected from the area which have undergone anagen induction and injection of fluva-gel 16 days post anagen induction, reveal HF in catagen/telogen without pigment and some pigmented HF in anagen (Figure 5a), while slides obtained from skin of mice after anagen induction and injection of placebo-gel exhibit dark HF in catagen/telogen (Figure 5b).

In slides of both groups there is no evidence of intensive lymphocytic infiltration after gel injections with fluvastatin or placebo.

**Figure 6:**
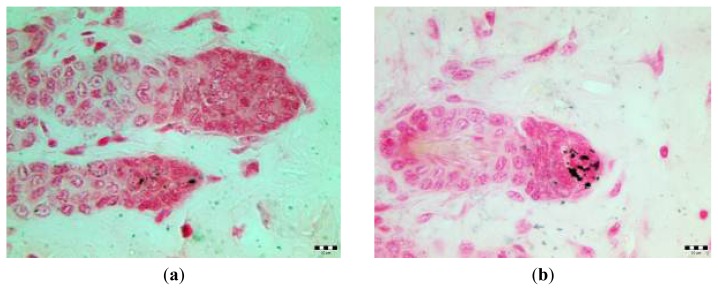
(**a**,**b**) Skin samples collected from anagen induction area of male mice 20 days after injection of fluva-gel 1 (**a**) and placebo-gel (**b**). Anagen induction was performed four weeks after birth. Fontana-Masson staining. Scale bar: 10 μm.

Stain for melanin (Fontana-Masson staining method) revealed almost lack of melanin in HF obtained from skin after anagen induction with injected fluva-gel 1 (Figure 6a) and granules of melanin in HF from the skin after AI and injection of placebo-gel (Figure 6b).

In the current paper we show for the first time that fluvastatin may influence hair color *in vivo*, as we have presented regrowth of light hair in C57Bl/6 mice after anagen induction and subcutaneous injection of gels containing fluvastatin (fluva-gel 1 and fluva-gel 2) (Figures 2a and 3), while there are no signs of depigmentation of hair in mice after injection of gel with placebo. We also demonstrate that expression of tyrosinase induced by UVB irradiation in B16F10 melanoma cell line was repressed in the presence of fluvastatin, which might partly confirm that fluvastatin effect of depigmentation is through the inhibition of melanin synthesis.

Effect of hair depigmentation after fluvastatin treatment was apparent only in some of the mice after injection of fluva-gel in various concentrations (Table 1: groups 1, 2, 3, 5). There were no macroscopic signs of depigmentation in mice without anagen induction after injection of fluva-gel 1 during sixth week of age (mice with HF in catagen/telogen) and during eight weeks of age (HF in telogen after first postnatal anagen) (Table 1: group 6 and 7, respectively) and there were no histopathological signs of inflammatory reaction in mice after AI and injection of fluva-gel 1 (group 2 in Table 1). Reduced number of grains of melanin in hair follicles collected from mice after fluva-gel (stain for melanin, Figure 6a) confirms that fluvastatin reduces melanin synthesis. Thus, results mentioned above, partly confirm that concentration of fluvastatin used in fluva-gel 1 is not toxic and that growth of depigmented hair is an effect of direct action of fluvastatin on melanin synthesis in the specific moment of hair cycle, the anagen. In the case of toxic action, light hair would be seen in all mice of that group. Furthermore, as presented on Figure 3, light hair at distal parts (first millimeters), were typically black, which also confirms that fluvastatin injection was not injurious and its effect was gradual. According to the previous investigations, decrease of tyrosinase synthesis might be simply a result of antiproliferative effect of statins resulting from their anticancerogenic effect [13–15]. Furthermore, statins, depending on cell type and pathological conditions, modulate via TGF-β important intracellular processes [16–18]; thus, decrease of melanin production may be caused by augmented degradation of tyrosinase, as observed in TGF-β1 treated B16F10 cell line [19].

Concentration of fluvastatin in fluva-gel 1 was calculated as 10 times the dose of 10 mg/kg/daily fluvastatin per adult C57Bl/6 male mice, weighing 27.2 ± 0.9 g. Daily doses of 10 mg/kg/daily fluvastatin per mice were used without side effects in other studies [20]. To achieve constant concentration of fluvastatin in the skin area, we used methylcellulose gel, whose properties guarantee minimal inflammation without apparent edema, haemorrhage, and necrosis due to subcutaneous single injection [21]. Thus, in all control groups (parts of group: 1–7) there were no signs of inflammation caused by methylcellulose gel injection. Average weights of young mice used in our experiments were below the average weight of adult mice for which we calculated concentration of fluvastatin, however the most important was comparable concentration of fluvastatin, as fluvastatin suspended in the gel acts locally. Exploration of dosage-dependent effect of fluvastatin injections was performed in four-week-old mice after anagen induction (not in seven-week-old mice), because frequency of depigmentation was higher in four week-old mice after AI and injection of fluva-gel 1 than in mice after AI performed in seven-week-old mice. Fluva-gel in the highest concentration of fluvastatin (fluva-gel 1) caused regrowth of depigmented hair in increased frequency, in comparison with effects caused by fluva-gel 2 and fluva-gel 3, and this result was statistically significant, indicating that the influence of gel with fluvastatin on melanin synthesis is dose-dependent.

Although hair pulled from hair follicle induces proliferation of certain cells of hair follicle and first postnatal telogen development (first spontaneous anagen) begins in the third week after birth in C57Bl/6 mice, to achieve fully synchronized anagen over the skin area, hair was removed also in four-week-old mice (in the first postnatal anagen) [22]. To verify whether fluvastatin injection might influence hair follicle during the first postnatal anagen, injections of fluvastatin gel (fluva-gel 1) were also performed in four-week old mice without depilation. In our research, injections of gel containing fluvastatin were performed at very early phase of anagen induction (first day after depilation), as hair follicles nine days after induction reach their morphological maturity. As wound healing occurs immediately after the depilation [23,24], effect of fluvastatin on the color of regrowing hair may be modulated by this process; particularly because statins have a potential to improve wound healing [25].

Our findings might have some clinical implications, thus such investigations should be extended in future clinical studies.

## 3. Experimental Section

### 3.1. Experiments *in Vitro*

#### 3.1.1. Cell Culture, Irradiation Procedure

B16F10 melanoma cells were kindly donated by Dr. Tomasz Stokłosa from Department of Immunology, Center for Biostructure, Medical University in Warsaw, Warsaw, Poland.

B16F10 mouse melanoma cells were cultured in 75 cm^2^ flask and grown in DMEM + GlutaMAX medium (Gibco, Grand Island, NY, USA) with 10% fetal bovine serum (Gibco, Grand Island, NY, USA) and 1% antibiotic/antimycotic solution (Gibco, Grand Island, NY, USA). Incubation was carried out at 37 °C under air-CO_2_ (95:5) atmosphere.

B16F10 cells were divided into four groups: cells irradiated with UVB and treated/not treated with fluvastatin and cells not irradiated and treated/not treated with fluvastatin, which served as controls. The source of UVB was a Cosmedico stimulator (Cosmedico Medizintechnik, Schwenningen, Germany), which emits energy in the UVB range, with the peak at 311 nm. Cells were irradiated twice (once daily (100 mJ/cm^2^), at 24 and 48 h of experiment) in PBS to avoid formation of medium-derived toxic photo-products [26]. Non-irradiated control cells were maintained in PBS during the time of irradiation procedure. Immediately after second irradiation, PBS was replaced with fresh warm growth medium with/without 1 μM fluvastatin (concentration of fluvastatin was determined by previously performed cell viability assay) [11]. Protein content measurements were done after 24 h following last (second) irradiation.

#### 3.1.2. Western Blot Analysis

Cells were lysed in RIPA buffer containing protease inhibitors (Complete, Roche, Mannheim, Germany) and centrifuged at 15,000× *g* for 30 min. The resultant solubilized proteins were measured using Bio-Rad DC Protein Assay, and equal quantities of each probe were subjected to sodium dodecyl sulfate-polyacrylamide gel electrophoresis (SDS-PAGE) on 8% polyacrylamide gel for tyrosinase. Proteins were transferred electrophoretically onto a PVDF membrane. Blocking was performed in Tris-buffered saline containing 5% skim milk powder and 0.05% Tween-20. Blots were incubated with the appropriate primary antibodies at a dilution of 1:1000 and then further incubated with horseradish peroxidase-conjugated secondary antibody. Membranes were washed in TBST buffer and proteins were detected by Western Blotting Luminol Reagent (Santa Cruz, CA, USA). Results were analysed using Bio-Rad (Hercules, CA, USA) GS-700 imaging densitometer.

Antibodies against: tyrosinase, β-actin, and secondary antibodies (HRP-goat anti-rabbit or HRP-bovine anti mouse) were purchased from Santa Cruz Biotechnology (Santa Cruz, CA, USA).

### 3.2. Experiments *in Vivo*

#### 3.2.1. Animals, Anagen Induction (AI), Fluvastatin Gel Injections, and Tissue Collection

One hundred ten C57Bl/6 mice were used (four-week-old: 31 female and 46 male mice; six-week-old: 6 male mice; seven-week-old: 7 female and 7 male mice; eight-week-old: 13 male mice; weighing respectively 10.0 ± 1.63 g; 10.86 ± 1.26 g; 16.6 ± 0.82 g; 15.67 ± 1.03 g; 19.75 ± 1.08 g; 21.08 ± 1.32 g). Institutional guidelines compliant to the Guiding Principles in the Care and Use of Animals, were adhered to, in accordance with the Medical University Ethics Committee guidelines for the care and use of laboratory animals. The mice were housed, up to five per cage, at 22 °C and 40% humidity under 12-h light/12-h dark cycle, with free access to water and normocholesterolemic mice chow (Animals, Pokarm podstawowy, Poland).

Animals were randomly divided into seven main experimental groups of mice, as presented in Table 1. Anagen induction (AI—active hair growth induction), in the first variant, was induced accurately with the method described previously in the first experimental group of mice (group 1 in Table 1) [27,28]. Briefly, a wax/rosin mixture was applied on the dorsal skin of anaesthetized seven-week old mice C57Bl/6 with all dorsal skin hair follicles in telogen, as evidenced by the homogeneous pink skin color. Mice were anaesthetized with an intraperitoneal injection of 0.07 mL solution of Rompun (Bayer AG, Leverkusen, Holland) and Calypsol (Richter Gedeon, Budapest, Hungary) in a 1:2 ratio. After removing the wax/rosin mixture, all hair shafts were peeled and immediately homogeneous anagen development was induced over the entire depilated back of the mouse. In the second variant of anagen induction, procedure has been modified: four-week-old mice were used (2nd, 3rd, and 4th group, as presented in Table 1). On the second day after AI, mice (groups 1–4) were anaesthetized again as described above, and 0.05 mL of 4.0% methylcellulose gel (4000 cps; Sigma-Aldrich, St. Louis, MO, USA) containing fluvastatin (fluva-gel) or gel alone (4.0% methylcellulose gel only)-placebo gel (placebo-gel) was injected subcutaneously over the dorsal depilated skin. Mice without AI (groups 5, 6, and 7 in Table 1) have also had an injection of fluva-gel or placebo-gel. Gel was prepared as described previously by Thylin *et al.*, except for statin used in this study: instead of simvastatin we used fluvastatin (Novartis, Basel, Switzerland) [29]. A 4.0% methylcellulose gel, which served as the placebo (placebo-gel) was previously prepared by adding 2 grams of polymer to 50 mL of hot distilled water, and was filled into a 1 mL syringes before cooling to gel at room temperature; a 4.0% methylcellulose gel as a vehicle for fluvastatin, before the formation of gel a proper amount of fluvastatin (as described below) was added, fluid was mixed and was filled into a 1 mL syringes and stored at the +4 °C. For exploration of dosage-dependent effect we prepared gels with various concentrations of fluvastatin. Addition of 2.7 g of fluvastatin per 50 mL of 4.0% methylcellulose gel allowed us to obtain a 2.7 mg of fluvastatin per volume of a single subcutaneous injection of adequate gel (50 μL), which made a tenfold higher daily dose of 10 mg/kg (fluva-gel 1) calculated for an average weight of adult C57/Bl6 mice (27 g); analogously we prepared gel with tenfold higher dose of 5 mg/kg (fluva-gel 2) and fluva-gel 3 with tenfold higher dose of 2.5 mg/kg. Hair follicles of four-week-old C57Bl/6 mice (group 5 in Table 1) are in first postnatal anagen, while HF of six-week-old mice are in catagen/telogen phase of hair cycle (group 6 in Table 1) and HF of eight-week-old mice (mice of the 7th group in Table 1) are in telogen after first postnatal anagen [28].

#### 3.2.2. Histology

After the experiment, under anaesthesia, skin of the back was shaved with an electrical animal clipper and washed briefly with 70% ethanol. Skin biopsies of three male mice selected randomly from second group (four-week-old mice after induction of anagen and injection of fluva-gel 1 and with regrowth of depigmented hair) were harvested 16 days after anagen induction, from the place where anagen was induced and gel was implanted. For control, skin biopsies were taken from anagen induction area of mice 16 days after injection of placebo-gel (also second group of mice). Afterwards, skin was sutured with 4–0 silk sutures.

Skin samples were fixed in Bouin solution and embedded in paraffin wax and 8 μm serial sections were stained with haematoxylin and eosin.

Skin samples for melanin staining (Fontana-Masson silver method) were collected 20 days following AI (16 days after injection of gel with fluvastatin) from the depilated area of mice injected with fluva-gel 1 and, for control, from AI area after injection of placebo-gel. Anagen induction was performed four weeks after birth. Stain was according to instruction of University of Nottingham Medical School, Division of Histopathology.

#### 3.2.3. Statistical Analysis

SAS V.6.12 for Windows software (SAS Institute, Cary, NC, USA) and SPSS 19 software (IBM SPSS Statistics, Armonk, NY, USA) was used for statistical data processing.

The differences between the levels of tyrosinase production measured by Western blot from four independent experiments were analysed using ANOVA test. The differences between the frequencies of depigmentation in female and male mice after anagen induction performed in four-week old mice and injection of fluva-gel (1–3) were evaluated using Fisher exact test. Statistical tests were considered significant when the *p* value was lower than 0.05. To determine the probability of depigmentation in the case of injection fluva-gel 1, fluva-gel 2, and fluva-gel 3 in those mice, a logistic regression analysis (LRA) was calculated.

## 4. Conclusions

Our study shows for the first time that fluvastatin might affect melanin synthesis *in vivo*. This is a very interesting and up to now unknown effect that could have clinical significance, as statins are used in a very large population of patients, including those with dermatological conditions. Whether such an effect should be considered while treating these patients, remains to be elucidated in further studies.

## Figures and Tables

**Table 1:** Randomization of C57Bl/6 mice and results of macroscopic examination of hair color after subcutaneous injections of gel with various concentrations of fluvastatin (fluva-gel 1–3) or injections of gel with placebo (placebo-gel).

Gender	Frequency of depigmentation % (number of mice with depigmentation after injection of fluva-gel/total number of mice after injection of respective fluva-gel)	Frequency of depigmentation % (number of mice with depigmentation after injection of placebo-gel/total number of mice after injection of placebo-gel)
Anagen induction in seven-week old mice—group 1		
fluva-gel 1		placebo-gel (control 1)
F	25 (1/4)	0 (0/3)
M	50 (2/4)	0 (0/3)
Anagen induction in four-week old mice—group 2		
fluva-gel 1		placebo-gel (control 2)
F	60 (3/5)	0 (0/3)
M	82 (9/11)	0 (0/7)
Anagen induction in four-week old mice—group 3		
fluva-gel 2		
F	25 (3/12)	
M	20 (2/10)	
Anagen induction in four-week old mice—group 4		
fluva-gel 3		
F	0 (0/8)	
M	0 (0/8)	
Mice without anagen induction:		
Four-week old mice in first postnatal anagen—group 5		
fluva-gel 1		placebo-gel (control 3)
F	0 (0/2)	0 (0/1)
M	37,5 (3/8)	0 (0/2)
Six-week old mice in catagen/telogen after first postnatal anagen—group 6		
fluva-gel 1 (control 4)		placebo-gel (control 5)
M	0 (0/4)	0 (0/2)
Eight – week old mice in telogen after first postnatal anagen—group 7		
fluva-gel 1 (control 6)		placebo-gel (control 7)
M	0 (0/8)	0 (0/5)

## Abbreviations

AI: anagen induction
HF: hair follicle
UVB: ultraviolet B
